# Annealing Response of Additively Manufactured High-Strength 1.2709 Maraging Steel Depending on Elevated Temperatures

**DOI:** 10.3390/ma15113753

**Published:** 2022-05-24

**Authors:** Angelina Strakosova, Filip Průša, Alena Michalcová, Petr Kratochvíl, Dalibor Vojtěch

**Affiliations:** Department of Metals and Corrosion Engineering, University of Chemistry and Technology, Prague, Technická 5, 166 28 Prague, Czech Republic; filip.prusa@vscht.cz (F.P.); alena.michalcova@vscht.cz (A.M.); petr.kratochvil@vscht.cz (P.K.); dalibor.vojtech@vscht.cz (D.V.)

**Keywords:** maraging steel, annealing response, mechanical properties, elevated temperatures, TEM-analysis

## Abstract

The present work describes the influence of different temperatures on mechanical properties and microstructure of additively manufactured high-strength 1.2709 maraging steel. For this purpose, samples produced by selective laser melting technology were used in their as-printed as well as their heat-treated state. Both samples were than exposed to temperatures ranging between 100 °C to 900 °C with a total dwell time of 2 h followed by water-cooling. The microhardness of the as-printed material reached its maximum (561 ± 6 HV0.1) at 500 °C, which corresponded to the microstructural changes. However, the heat-treated material retained its initial mechanical properties up to 500 °C. As the temperature increased, the microhardness of both the materials reduced, reaching their minimum at 900 °C. This phenomenon was accompanied by a change in the microstructure by forming coarse-grained martensite. This also resulted in a significant decrease in the ultimate tensile strength and an increase in the plasticity. TEM analysis confirmed the formation of Ni_3_Mo intermetallic phases in the as-printed material when exposed to a temperature of 500 °C. It was found that the same phase was present in the heat-treated sample and it remained stable up to a temperature of 500 °C.

## 1. Introduction

In general, maraging steel is highly alloyed with Ni, Co, Mo, and Ti. They have a very low carbon content (usually up to 0.03 wt.%) which allows them to achieve high strength and good toughness [1,2]. The combination of such good mechanical properties is possible due to precipitates formed in the soft martensite structure after the heat treatment as an integral part of the processing [3,4,5,6,7,8]. The 1.2709 is a type of high-strength maraging steel that combines outstanding mechanical properties (hardness around 55 HRC, ultimate tensile strength up to 2000 MPa), toughness, good weldability, machinability, and spatial stability during the heat treatment process. These properties are achieved due to the formation of Ni_3_Mo, Ni_3_Ti, and Fe_2_Mo nanoprecipitates during heat treatment consisting of solution annealing and subsequent aging [5,6,7,8,9,10]. This type of material is widely used in the automobile and aerospace industries, and in weapon and tool production [7]. Maraging steel is therefore an attractive material that can be produced by a variety of technologies from conventional ones [1,2,3,5] to modern additive manufacturing (AM) methods [4,6,7].

AM technology, also known as 3D printing, is nowadays used in different areas: medicine, aerospace, architecture, science, and many others [11]. In addition, its attractiveness further increases because of its ability to be used for a wide range of materials starting from polymers and ceramics up to high-strength steels [11]. Also, AM technology allows the production of components with an intricate and precise shape in the range of microscopic to macroscopic size, reducing the number of machining operations of the finished product and the waste content. As-built components are characterized by a very fine microstructure, which contributes to increasing the values of mechanical properties. These are the advantages of AM compared to classical methods. The main drawbacks are a small batch of samples produced during one printing process and a relatively low building rate.

It is well known that aging at temperatures from 400 °C to 650 °C increases the maraging steel hardness [10]. It happens due to the strengthening effect of different types of nanoscaled intermetallic phases. At lower temperatures (400–480 °C), the Ni_3_Mo and Ni_3_Ti-phases usually form [12,13,14]. However, higher temperatures allow the formation of Fe_2_Mo precipitates [15,16,17]. Therefore, many researchers have described the microstructure and mechanical properties of maraging steel as a product of different aging temperatures [3,4,7]. Some studies [6,8,12,14] are related to the two-stage heat treatment investigation of the maraging steel, where the solution annealing at temperatures between 815–950 °C has been used before the aging step. Although the aging response of the material has been reported many times [18,19,20,21], there is almost no information about the annealing response of maraging steel 1.2709 prepared by 3D printing or a combination of 3D printing and subsequent heat-treatment [22]. This information would be beneficial for the evaluation of the thermal stability of the 3D-printed steel.

The present work aims to investigate how the 3D-printed high-strength 1.2709 maraging steel behaviour after being exposed to elevated temperatures. Therefore, the microstructure and mechanical properties of the 3D-printed and 3D-printed plus heat-treated maraging steel were described as a function of annealing at temperatures ranging from 100 °C up to 900 °C.

## 2. Materials and Methods

High-strength 1.2709 maraging steel (also denoted as X3NiCoMoTi 18-9-5) was investigated in this work. The 3D printed steel samples were purchased from a commercial partner and produced using a Selective Laser Melting (SLM) technology, for which the parameters are part of the company know-how.

The as-printed material was cut into 20 samples of 13 × 5 × 3 mm in size using a precision metallographic cut-off ATM Brillant 220 (ATM GmbH, Mammelzen, Germany) machine. Half of these samples were then heat-treated (hereafter denoted as as-heat-treated) in an electric resistance furnace. The remaining ones were tested in the as-printed state (hereafter denoted as as-printed). The heat treatment regime (hereafter denoted as SAT) consisted of the following steps: 1. solution annealing (820 °C/1 h), 2. air-cooling to the room temperature (RT), 3. artificial aging (490 °C/6 h) and 4. air-cooling to RT [18]. 

Both the sample types were then tested for their annealing response by exposing them to elevated temperatures ranging from 100 °C to 900 °C with a total dwell time of 2 h, followed by water-cooling. The phase composition of the samples was determined using the X-ray diffraction spectroscopy (XRD) PANalytical X’Pert Pro (Almelo, The Netherlands) with Co-Kα source (λ = 0.17929 nm). These measurements were done in a 2θ range of 6–110° with a step size of 0.039° and a scan step time of 175.185 s. Differential thermal analysis (DTA) Setsys Evolution (Setaram, Sophia Antipolis, Francie) was used to study the ongoing processes in the as-printed and as-heat-treated samples during heating. The parameters of the analysis comprised a heating rate of 10 °C/min up to a temperature of 1000 °C in the Ar atmosphere.

Mechanical properties were studied by Vickers microhardness tests (load of 0.1 kg, HV0.1) and tensile tests. Microhardness was measured on a FUTURE TECH FM-700 testing machine (FUTURE-TECH CORP., Kawasaki-City, Japan) using at least 10 indentations for each sample to obtain a statistical relevance. Tensile tests were performed at laboratory temperature on as-printed samples with a dog-bone shape, having total L × W × H dimensions of 68 × 7 × 3 mm and a tested section L × W × H of 28 × 5 × 3 mm, both shown in detail in the study [18], with a constant strain rate of 0.001 s^−1^ using the universal testing machine LabTest 5.250SP1-VM (LaborTech, Opava, Czech Republic). The microstructure was observed on the metallographic samples using a scanning electron microscope (SEM) TESCAN LYRA3 (Brno, Czech Republic). For this purpose, samples were ground (SiC abrasive paper with grit size P240-P4000), polished (diamond paste and Eposil F suspense), and etched in a Nital 2 reagent (2 mL HNO_3_ + 98 mL ethanol). For the detailed microstructure analysis, a transmission electron microscope (TEM) JEOL 2200 FS (JEOL, Akishima, Japan) equipped with an energy dispersive spectrometer (EDS) (Oxford Instruments, 80 mm^2^, High Wycombe, UK) were used. 

## 3. Results and Discussion

### 3.1. Mechanical Properties

**Figure 1:**
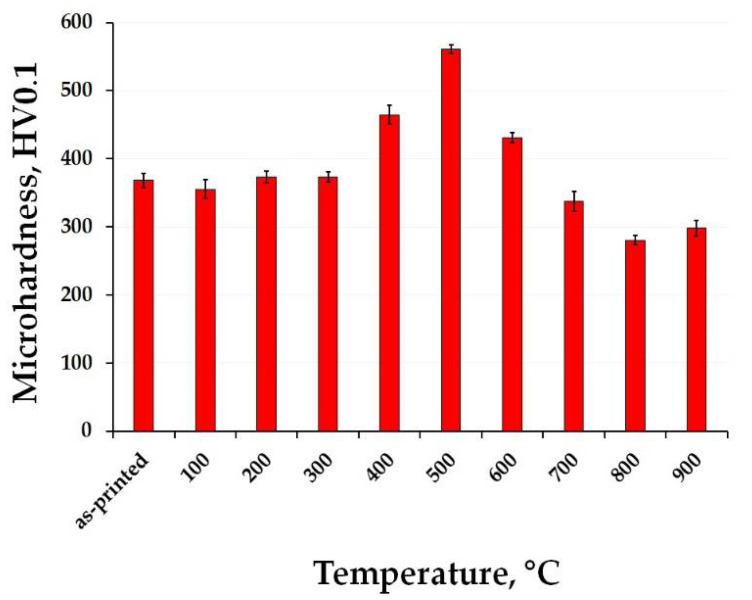
Microhardness of the as-printed 1.2709 maraging steel as a response to exposure to different temperatures with a total dwell time of 2 h.

The microhardness change of the as-printed 1.2709 maraging steel as a product of expositions to elevated temperatures is shown in Figure 1. It can be seen that the as-printed steel is a relatively soft material with Vickers microhardness values of around 370 HV0.1. These values remain almost the same during annealing up to 300 °C/2 h, indicating that the steel microstructure did not change significantly at these temperatures. At the temperature of 400 °C, the microhardness starts to increase, achieving its maximum of approximately 560 HV0.1 at 500 °C. A further temperature increase results in hardness reduction to a minimum below 300 HV0.1 especially when annealed at 800 °C or higher temperatures. This hardness is even lower than that of the as-printed steel.

The temporary microhardness increase (Figure 1) of the as-printed material after annealing at moderate temperatures was also observed in the work of Floreen et al. [10] confirming the formation of nanosized intermetallic precipitates within this temperature range. Up to 300 °C, the as-printed steel remains a relatively soft material due to the absence of these reinforcing particles. However, in the range of 400–500 °C, the newly forming precipitates lead to a strengthening of the maraging steel. Nevertheless, exceeding the temperature 500 °C causes a rapid growth of precipitates, increases of their average interparticle distance, and slow dissolution which is accelerated with the temperature. Both processes are known for decreasing the microhardness while simultaneously increasing the ductility of the steel. Similar observations were reported in recent studies on the maraging steel [4,23,24] and are in good correlation with the hardness increase reported in the present work reaching the maximum of 561 ± 6 HV0.1 at 500 °C due to the formation of precipitates which strengthened the alloy.

**Figure 2:**
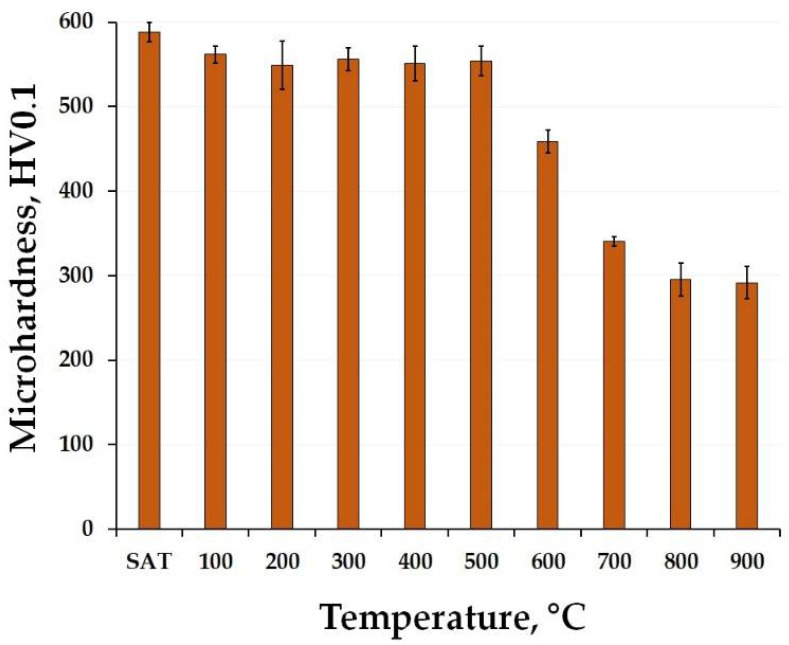
Microhardness of the as-heat-treated 1.2709 maraging steel as a response to exposure to different temperatures with a total dwell time of 2 h.

On the other hand, the microhardness of the as-heat-treated material (Figure 2) shows a different trend. It only slightly decreases after exposure at 100 °C and than remains almost constant up to 500 °C. This confirms that the phases formed during the heat treatment (solution annealing and aging) were thermally stable up to this temperature. This trend is similar to that observed in the case of as-printed material, which reached its maximum at 500 °C. From this point of view this confirms the above-stated fact, that the state of processing of such material is extremely important since it determines the consequential mechanical behavior during a relatively wide range of temperatures. In contrast, higher annealing temperatures caused a decrease in microhardness reaching its minimum below 300 HV0.1 when annealed at 900 °C. 

The decrease in observed microhardness at temperatures above 500 °C reflects other microstructural changes at these temperatures. It can be assumed that annealing at 600 °C and more causes the beginning of the austenite transition of the steel, which is in a range of 622–642 °C [25], either in part or in total volume. The same effect of the annealing at 800 °C and 900 °C can be associated with the fact that those temperatures are high enough to fully austenitize the steel and form a martensite microstructure after cooling. According to the work of Król et al. [25], the final temperature of austenite formation was reported to be found between 818–825 °C, which is slightly above the 801 °C mentioned in [26]. Austenite undergoes diffusionless martensitic transformation upon cooling to RT. Due to very low carbon and high Ni content, the martensite is characterized by a low hardness, similarly to initial austenite [2,6]. Moreover, rapid coarsening and partial dissolution of precipitates can be expected at above 500 °C which also contributes to the softening of the steel.

**Figure 3:**
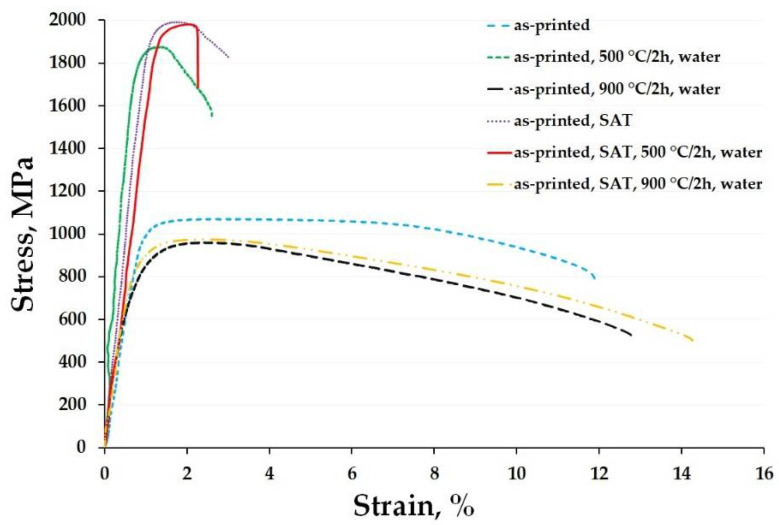
Tensile tests of the 1.2709 maraging steel under a variety of conditions including the initial state (as-printed, and as-printed, SAT) and those which were annealed at 500 °C and 900 °C for 2 h and water quenched.

The tensile stress-strain curves of the studied materials are shown in Figure 3. One can see that the as-printed material reached ultimate tensile strength (UTS) value above 1000 MPa while retaining good ductility. When annealed at 500 °C/2 h, the tensile yield strength (TYS) and UTS values increased rapidly as the ductility decreased. The opposite effect was caused after exposing the material to the highest temperature of 900 °C/2 h. The strength properties significantly deteriorated, falling below 1000 MPa, and showing a slight increase in ductility. This trend corresponds to the microhardness changes observations (Figure 1) where the as-printed material exhibited the highest values when annealed up to 500 °C, and started to soften after exceeding that temperature. In comparison, the SAT heat-treated material showed the highest UTS value at the expense of a significant decrease in elongation. After the as-heat-treated material was exposed to 500 °C, it caused only a minor decrease in the properties. However, when it was annealed at 900 °C, the features changed significantly, having almost the same performance as the as-printed sample annealed at 900 °C. The above-mentioned results can be matched for both the tested types of materials with the observed microhardness changes (Figure 2).

Exposing the materials to temperatures up to 500 °C positively influenced the mechanical properties due to generally known precipitation hardening. Here, the matrix of the material depletes some alloying elements (usually Ni, Ti, Mo) which form Ni_3_(Mo, Ti) intermetallic phases decreasing the ductility. But there are no evident phase composition changes (see below). The decrease in mechanical properties and increase in ductility of material exposed to 900 °C can be justified by the fact that this temperature is higher than the austenite transition finish temperature [25,26]. This means that exposure at 900 °C causes full dissolution and homogenization of austenite structure which transforms into martensite after cooling to room temperature (see below).

### 3.2. *Differential Thermal Analysis* (DTA)

**Figure 4:**
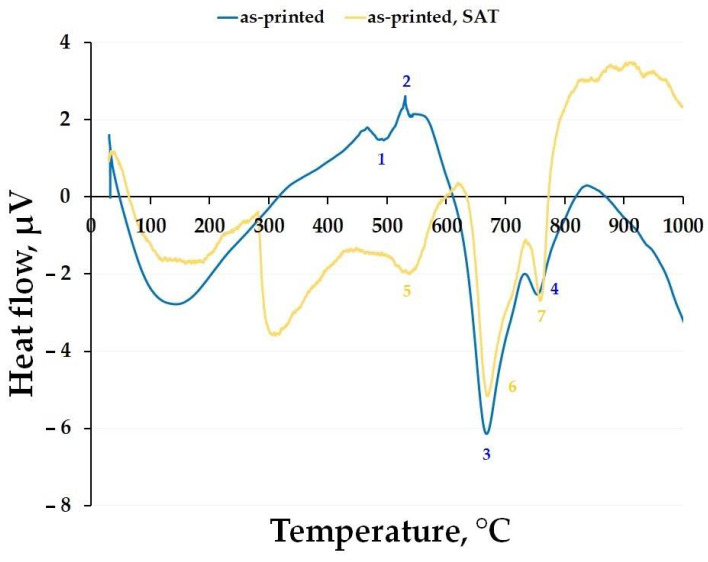
DTA curves for 1.2709 maraging steel depending on the heat treatment (identified peaks are labeled with numbers).

The DTA of the maraging steel was done to describe the material’s behaviour at elevated temperatures (Figure 4). One can observe that four peaks appeared in the as-printed material during heating, compared to the as-heat-treated one, where only three peaks appeared. The first endothermic peak (1) is believed to be assigned to the formation of the precipitates which are responsible for the hardening of the steel. This can be confirmed by the mechanical testing (Figure 1 and Figure 3) of the material, where heating at 500 °C causes a rapid increase in the microhardness and the UTS. The second exothermic peak (2) can be attributed to the beginning of the coarsening of the precipitates and grain growth of the retained austenite [27]. Peaks 3 and 4 are endothermic; they are responsible for the precipitate dissolution and for the transformation of the martensite to the austenite phase, which starts at temperatures higher than 600 °C [23,25,26]. This phenomenon is supported by the decrease in the microhardness of the as-printed material when it is annealed at temperatures higher than 500 °C (Figure 1). 

The DTA curve of the as-heat-treated material exhibits three endothermic peaks. The first of them (peak 5) can be attributed to the increasing size of the precipitates and the distances between them. Peaks 6 and 7, similar to the peaks 3 and 4 in the as-printed sample, correspond to phase transformation to the austenite resulting in a mechanical properties decrease (see Figure 2). The endothermic peak onsetting around 300 °C in the case of the as-heat-treated sample corresponded to a sudden endothermic deflection from the drifting baseline caused primarily by the machine itself. Therefore, this peak was not considered a material related response towards the slow heating up during DTA analysis.

### 3.3. X-ray Diffraction (XRD)

**Figure 5:**
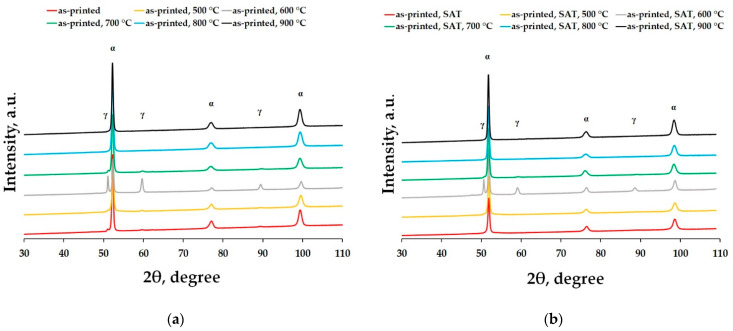
XRD patterns of the 1.2709 maraging steel depending on elevated temperatures: (**a**) as-printed; (**b**) as-heat-treated (SAT—solution annealing and aging treatment); holding time 2 h, water-cooling.

To confirm the phase transformations as a product of annealing, XRD analysis of the studied samples has been performed. One can see that the as-printed (Figure 5a) and the as-heat-treated (Figure 5b) materials have the same behaviour in terms of phase transformations at the same temperatures. 

It can be seen that the annealing temperature of 500 °C does not affect the phase composition changes of the maraging steel. Therefore, we can say that it is responsible for the precipitation hardening, manifesting itself by a rapid increase of the mechanical properties’ values (Figure 1, Figure 2 and Figure 3). The biggest changes were caused by the material heating at a temperature of 600 °C. Here we can see the formation of the gamma phase (or retained austenite), which is a soft phase. Moreover, this temperature is high enough to cause coarsening or, on the top of it, extinction of the precipitates (usually Ni_3_Mo or Ni_3_Ti). This means that the matrix of the steel begins to enrich itself with Ni which expands the austenite stability area and makes the martensite a soft phase. Therefore, one can say that this temperature can be responsible for the beginning of the microhardness decrease observed in both the studied materials (Figure 1 and Figure 2). In Figure 5 it can be seen that a temperature of 700 °C is not enough to obtain a fully homogeneous microstructure. Therefore, the material still contains a small percentage of retained austenite which completely disappears at temperatures exceeding 800 °C. The XRD spectra confirm the abovementioned DTA analysis results which showed the existence of endothermic peaks corresponding to phase transformation onsetting around 600 °C in both the tested materials (see Figure 4).

### 3.4. Microstructure Characterization

**Figure 6:**
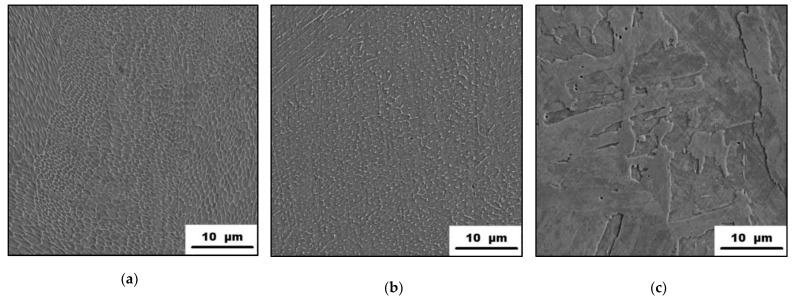
Microstructure of the 1.2709 maraging steel in different states showing: (**a**) as-printed; (**b**) as-printed, 500 °C/2 h; (**c**) as-printed, 900 °C/2 h materials (SEM).

The microstructures of the as-printed maraging steel in its initial state and after being exposed to 500 °C and 900 °C are shown in Figure 6. It is visible that the as-printed steel (Figure 6a) has a very fine microstructure that consists of cells. These cells started to disappear during their exposure to 500 °C for 2 h (see Figure 6b). This effect is even more significant when exposed at 900 °C, transforming the fine cell microstructure into the coarse-grained one (Figure 6c). The appearance of the as-printed microstructure can be explained by the manufacturing process itself. The selective laser melting process can be briefly described as the highly localized melting of small amounts of powder feedstock material. For such a process, a high energy of laser beam is needed, creating an enormous thermal gradient within the material, allowing rapid heat dissipation. Typically, the cooling rates are locally achieving up to 10^6^ K/s [27,28] which allows the formation of a cellular morphology typical for rapidly solidified alloys. As the maraging steel is exposed to 500 °C/2 h, the precipitation with the Ni_3_(Mo,Ti)-phases takes place. This means that the material volume depletes itself by the precipitate-forming elements and this can cause the beginning of the dissolution of the cells’ boundaries. However, such precipitates cannot be observed by SEM in Figure 6b. Finally, exposing the as-printed material to a temperature of 900 °C resulted in homogenization, cell disappearance, and the formation of a coarse-grained microstructure (Figure 6c). Since the temperature of 900 °C is above the austenite transition finish temperature, which is in a range of 818–825 °C [25], the lath martensite is observed in this sample after the water-cooling to RT (Figure 6c). These microstructure changes are in good agreement with the XRD-analysis results (Figure 5a).

**Figure 7:**
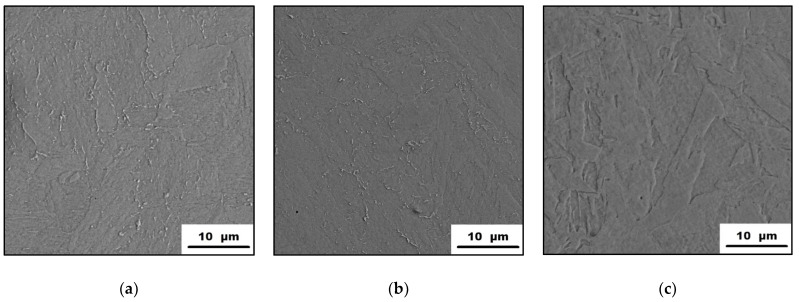
Microstructure characterization of the as-heat-treated 1.2709 maraging steel depending on the temperature: (**a**) as-printed; (**b**) as-printed, 500 °C; (**c**) as-printed, 900 °C (SEM).

The microstructures of the as-heat-treated 1.2709 maraging steel in different states are shown in Figure 7. It can be seen that the as-heat-treated material is composed of lath martensite structures (Figure 7a), which is a consequence of the heat treatment regime composed of solution annealing at 820 °C, allowing microstructural homogenization and the formation of martensite microstructure. The consequential aging at 490 °C resulted in the formation of precipitates within the material, increasing the hardness of the material, which was found to be retained even when further exposed to temperatures up to 500 °C (Figure 2). These microhardness values were comparable to those of as-printed material being exposed only at 500 °C for 2 h (Figure 1). The microstructure and phase composition of the studied material did not change even when exposed at 500 °C (Figure 7b). It means that the microstructure of the 1.2709 maraging steel after solution annealing and aging treatment is stable up to 500 °C. The effect of the 900 °C on the microstructure is more distinctive than the previous one. It can be seen (Figure 7c) that the relatively fine martensitic structure (Figure 7a,b) has transformed into the coarser one, and has become similar to the microstructure of the as-printed material exposed at 900 °C (Figure 6c). The formation of such a coarse martensitic microstructure was caused by the high temperature and the holding time of the annealing that induced phase composition changes (Figure 5b) affecting the mechanical properties (Figure 2 and Figure 3).

Based on the microhardness response to annealing, the steel samples annealed at 500 °C/2 h were selected for detailed structural examination. The temperature of 500 °C represent the hardness maximum for the as-printed material and a limit above which hardness drop is observed for both as-printed and as-heat treated steel, see Figure 1 and Figure 2. 

**Figure 8:**
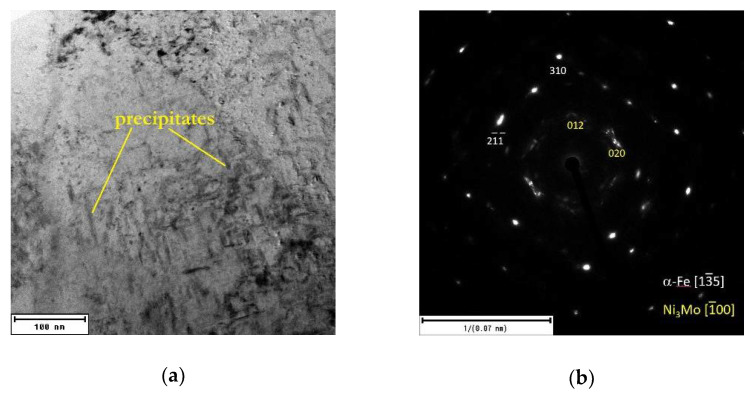

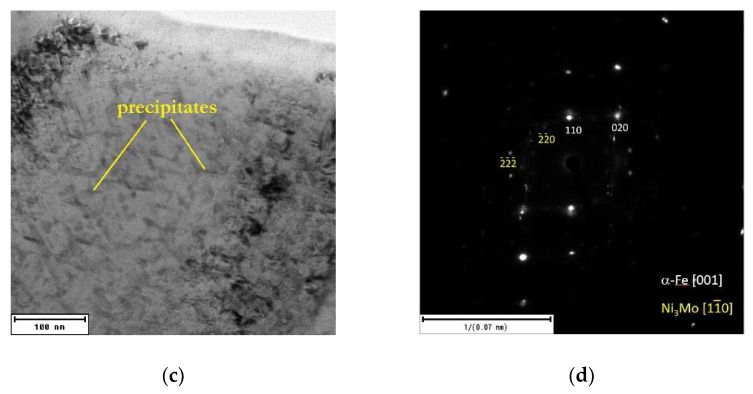
TEM micrographs and SAED patterns of the 1.2709 maraging steel after heating at 500 °C: (**a**,**b**) as-printed; (**c**,**d**) as-heat-treated.

Figure 8 shows the detailed TEM micrographs of both the materials after exposure at 500 °C for 2 h. It is obvious (Figure 8a,c) that rod-like precipitates grew up in both materials. These precipitates were identified by the SAED analysis (Figure 8b,d) as Ni_3_Mo intermetallic phases. This confirmed the fact that the temperature of 500 °C has only a hardening effect by the nanoscaled precipitations in the as-printed material (Figure 1 and Figure 3). However, this temperature was not high enough to induce phase transformation or precipitate dissolution in the as-heat-treated material, which can be justified by the DTA analysis (Figure 4).

The obtained results of the TEM analysis can be used to justify the previously described changes in structure and mechanical properties. It was found that the as-printed steel is a relatively soft material with a very fine cell structure. This changes when exposed at 500 °C for 2 h, which affects the formation of the precipitates as the cell boundaries begin to disappear. This positively affects the mechanical properties increasing the HV0.1, TYS, and UTS values. It can be justified by the fact that the Ni, which is responsible for the soft martensite phase in maraging steels, started being depleted from the martensitic phase due to the formation of strengthening Ni_3_Mo precipitates within the material volume. As for the as-heat-treated material, its mechanical properties had the highest values of HV0.1, TYS, UTS, and even elongation compared to other samples when exposed at elevated temperatures. This was caused by the solution annealing which caused homogenization and elimination of internal stresses within the microstructure. The consequential aging affected the formation of the precipitates, resulting in material hardening. The as-heat-treated material, which was exposed at 500 °C for 2 h and then water-cooled, has shown only a slight microhardness change (see Figure 2) which confirms a good annealing response up to these temperatures.

## 4. Conclusions

This work reports an annealing response of additively manufactured high-strength 1.2709 maraging steel when exposed to elevated temperatures. For this purpose, two types of starting material were used, including the as-printed material and the as-heat-treated material. The results of deliberate exposure to elevated temperatures ranging from 100 °C to 900 °C can be summarized as follows:

- The microhardness of the as-printed material started to increase at 400 °C and reached its highest value when exposed at 500 °C for 2 h. This value corresponded to the microhardness of the as-heat-treated material (solution annealed at 820 °C/1 h and aged at 490 °C/6 h). On the other hand, this maximum hardness was retained in the as-heat-treated material throughout the entire temperature interval up to 500 °C. Reaching a temperature of 900 °C, both materials significantly softened, decreasing their microhardness to approximately 300 HV0.1.
- The tensile stress-strain tests showed that the as-printed material significantly strengthened during exposure at 500 °C. However, the TYS and UTS values were slightly lower (≈200 MPa) compared to those of the as-heat-treated material.
- Increasing the temperature beyond 500 °C produced a coarsening of each present microstructural component, resulting in the formation of coarse-grained martensite and the overall softening of both the investigated materials, which was confirmed by the DTA and XRD analysis.
- TEM analysis has confirmed that, after annealing at 500 °C with a holding time of 2 h, the formation of Ni_3_Mo intermetallic phases takes place in the as-printed samples. Furthermore, the same phases were present in the as-heat-treated sample and remained stable up to 500 °C/2 h.

Due to the fact that the studied maraging steel is used in a heat-treated state, applications with an operating temperature above 500 °C are the main limitations of the material use.

## Data Availability

Data is contained within the article.
